# Serological testing for Lyme Borreliosis in general practice: A qualitative study among Dutch general practitioners

**DOI:** 10.1080/13814788.2020.1732347

**Published:** 2020-03-11

**Authors:** Tjitske M. Vreugdenhil, Mariska Leeflang, Joppe W. Hovius, Hein Sprong, Jettie Bont, C. W. Ang, Jeanette Pols, Henk C. P. M. Van Weert

**Affiliations:** a Department of General Practice, Amsterdam UMC/University of Amsterdam, Amsterdam, Netherlands;; b Department of Clinical Epidemiology, Amsterdam UMC/University of Amsterdam, Amsterdam, Netherlands;; c Department of Internal Medicine, Amsterdam UMC/University of Amsterdam, Amsterdam, Netherlands;; d Centre for Infectious Disease Control, National Institute for Public Health and Environment, Bilthoven, Netherlands;; e Department of Medical Microbiology and Infection Control, Amsterdam UMC/VU Medical Centre, Amsterdam, Netherlands

**Keywords:** Infectious diseases, general practice, family medicine, quality of care, qualitative designs and methods

## Abstract

**Background:** Concerns are raised about missed, delayed and inappropriate diagnosis of Lyme Borreliosis. Quantitative descriptive studies have demonstrated non-adherence to the guidelines for testing for Lyme Borreliosis.

**Objectives:** To gain insight into the diagnostic practices that general practitioners apply for Lyme Borreliosis, their motives for ordering tests and how they act upon test results.

**Methods:** A qualitative study among 16 general practitioners using semi-structured interviews and thematic content analysis.

**Results:** Five themes were distinguished: (1) recognising localised Lyme Borreliosis and symptoms of disseminated disease, (2) use of the guideline, (3) serological testing in patients with clinically suspect Lyme Borreliosis, (4) serological testing without clinical suspicion of Lyme Borreliosis, and (5) dealing with the limited accuracy of the serological tests. Whereas the national guideline recommends using serological tests for diagnosing, general practitioners also use them for ruling out disseminated Lyme Borreliosis. Reasons for non-adherence to the guideline for testing were to reassure patients with non-specific symptoms or without symptoms who feared to have Lyme disease, confirmation of localised Lyme Borreliosis and routine work-up in patients with continuing unexplained symptoms. Some general practitioners referred all patients who tested positive to medical specialists, where others struggled with the explanation of the results.

**Conclusion:** Both diagnosis and ruling out of disseminated Lyme Borreliosis can be difficult for general practitioners. General practitioners use serological tests to reassure patients and rule out Lyme Borreliosis, thereby deviating from the national guideline. Interpretation of test results in these cases can be difficult.

KEY MESSAGES

- GPs sometimes test to confirm or reject erythema migrans or disseminated Lyme disease, but are not always aware of possible false positive or negative results and tend to refer in case of an unexpected test result.
- Some GPs consider diagnosis of disseminated Lyme Borreliosis not their task.

## Introduction

With increased incidence of tick bites and Lyme Borreliosis (LB) in the Netherlands and other parts of Europe [1,2], public concern has come up about diagnostic practices for LB. On the one hand, there are concerns about missed and delayed diagnoses of LB. On the other hand, there are worries about incorrect diagnosis of LB, which may cause distress and treatment-related illness [3,4]. As gatekeepers of the health care system, Dutch general practitioners (GPs) are the first to be approached by patients with questions regarding LB. In the Netherlands, early-localised LB is a rather common disease whereas disseminated LB rarely occurs [5]. Early localised LB often presents with a typical annular rash, Erythema Migrans (EM), and occasionally with a Borrelial lymphocytoma. Early localised LB may also pass unnoticed [6]. Characteristic manifestations of disseminated LB are facial nerve palsy, meningo-radiculitis, arthritis and less frequently carditis, acrodermatitis chronica atrophicans and ocular symptoms [6]. Non-specific symptoms like fatigue, myalgia, arthralgia and fever can occur in localised and disseminated LB [7], but also occur frequently without objective signs of LB.

Diagnosis of LB is straightforward in patients with classic EM, as the typical annular rash is pathognomonic. Recognising disseminated LB is more difficult because it may mimic other more frequently occurring diseases, for example, Bell’s palsy. Diagnosis of disseminated LB is based on clinical symptoms and a history of a tick bite, confirmed by serological testing. The accuracy of the commonly used serological tests depends on the stage of the disease. The sensitivity ranges from approximately 50% in patients with EM to 77% in patients with neuroborreliosis and 97% in patients with acrodermatitis chronica atrophicans [6]. The specificity is approximately 80% in clinical practice and 95% in healthy controls, due to cross-reactivity and persisting antibodies after a resolved Borrelia infection. Discrimination between active and resolved infections based on test results may be difficult [8].

A national guideline for diagnosis and treatment of LB was published in the Netherlands in 2013 [7]. This guideline guides ordering serological tests based on the estimated pre-test probability of LB. Serological testing should be considered in patients with a high, intermediate or low probability of disseminated LB and should not be done in patients with a very low probability. Thus, patients with characteristic symptoms of disseminated LB, for example, arthritis or facial nerve palsy, and a history of a tick bite should be tested. Serological testing may be considered in patients with characteristic symptoms without a tick bite and patients with non-specific symptoms with a tick bite because the probability of LB is respectively intermediate and low [7]. The guideline does not recommend serological testing in patients with non-specific symptoms without a history of tick bite or in a-symptomatic patients. In these cases, the pre-test probability of LB is very low, and the risk of false-positive test results is substantial. Serological testing in patients with a typical EM is also not recommended because a negative result does not rule out LB and a positive result just confirms a diagnosis that was already clear based on the presence of EM [8]. The Dutch College of General Practitioners suggests that serological testing for LB should not be done in general practice because interpretation of test results may be difficult for GPs [9].

There is evidence that Dutch GPs, like physicians in other countries [10–12] do not adhere to the recommendations for ordering serological tests for LB [3,13]. Two recent studies demonstrated that Lyme serology is frequently requested in patients with non-specific symptoms [3,13]. This suggests over-testing, which carries a risk for inappropriate diagnosis and treatment of disseminated LB. The two studies also reported that serological testing was ordered in patients with EM, patients with locally irritated tick bites and a-symptomatic tick bites [13]. However, these studies did not investigate motives for ordering the serological tests, nor did they assess how was acted upon test results.

The public concerns about missed and delayed diagnosis of LB and the suggestion of over-testing and potential inappropriate diagnosis or inappropriate ruling out of LB indicate that further research is needed about the diagnostic practices for LB. This study aimed to gain insight into the diagnostic practices that Dutch GPs apply regarding LB. 1) What strategies are used by GPs when patients present with symptoms of LB or with questions about LB? 2) What reasons do GPs have for ordering serological tests for LB? 3) How do they interpret test results and act upon them, when testing was ordered against the guideline recommendations?

## Methods

### Study design

A qualitative study was conducted among Dutch GPs. A purposeful sampling strategy was applied to obtain variation among the GPs in gender, age, clinical experience, practice form, estimated socioeconomic status of patient population, affiliation to a university as supervisor of resident GPs and incidence of tick bites and EMs in the area of practice, based on reported local incidences of EM in 2014 [14]. GPs were recruited through contacts of the department of general practice of the Academic Medical Centre in Amsterdam. Most of the approached GPs agreed to participate; five were not interested. The sample size was determined by saturation, i.e. recruitment was ended when three consecutive interviews did not provide new information.

All interviewed GPs were informed on beforehand on the purpose of the study, i.e. to investigate how GPs acted when confronted with (questions on) possible Lyme disease and were asked to search in their files for patients, that had consulted.

#### Data collection

GPs were interviewed, using a semi-structured approach. The first author, a GP trained in interview techniques for qualitative research, conducted all interviews. All interviews started with an open question about the GPs’ experiences with LB in their practices and their use of serological testing for LB. Then they were asked to describe cases of patients with possible LB (related) problems or questions about LB or testing for LB. Also, a topic list was used including ‘clinical manifestations for LB including specific and non-specific symptoms’, ‘serological testing’, ‘interpretation of test results’, ‘treatment’, ‘referral to medical specialists’, ‘consultation of medical specialists’ and ‘LB guidelines’. After six interviews, the complete research team met and decided not to adjust the topic list but to ask more in-depth questions about consultation of and referral to medical specialists, as this was crucial to learn about the patients’ trajectory.

### Analysis

Each interview was transcribed verbatim. All interviews were read and coded by the first author and by at least one other member of the research team. Thematic content analysis was done using MAXQDA software. First text fragments were coded according to the themes of the topic list. Sub-codes of the clinical manifestation codes were created for text fragments on described cases of LB, testing strategies, interpretation of results, referral, patients’ preferences or demands. In an iterative process, new codes were added for testing on patients’ request, testing as part of routine blood analysis, guideline adherence. Themes were discussed with members of the research team.

## Results

Sixteen GPs were interviewed, of whom eight worked in regions with a lower incidence of EM than the nationwide incidence. The other eight GPs worked in areas with a higher incidence of EM than the national incidence. The characteristics of the 16 GPs that were interviewed are presented in Table 1. All GPs were aware of the existence of the Lyme Borreliosis guideline at the time of the interview. We distinguished five themes concerning diagnostic and testing practices for LB.

**Table 1.** Characteristics of GPs (*N* = 16).

Characteristic	Variables	Number
Gender of patients	Male	9
	Female	7
Years of clinical experience of GP	≤10 years	7
	>10 years	9
Practice form of GP	Health centre (GPs and health worker from other disciplines)	6
	Single handed/2 GP practice	5
	3 or more GP practice	5
SES of population (estimated by the GP)	Mainly lower SES	4
	Mainly higher SES	4
	Mixed SES	8
Affiliation with Department of General Practice of University as supervisor of GP residents or medical students	Affiliated	10
	Not affiliated	6
Risk of tick bites or EM in practice area based on reported local incidences of EM in 2014 (nationwide incidence of EM 140/100.00)	Lower than nationwide risk (EM incidence ≤140/100.000)	8
	Higher than nationwide risk (EM incidence >140/100.000)	8

### Recognising EM and symptoms of disseminated LB

The GPs in our study usually recognised EM themselves. Disseminated LB was not always recognised or considered by the GP. However, patients with severe or persisting unexplained symptoms were referred to medical specialists for further analysis.

‘An older lady with a headache, severe pain, no neurological deficits. I had not thought of LB. (…) The neurologist diagnosed Borrelia meningitis. Apparently, she also had something with the facial nerve, but I had not noticed that at the time.’

Similarly, rarer manifestations of LB were not always recognised, but were referred to medical specialists for diagnosis.

*(*About a case of acrodermatitis atrophicans*) ‘Yes, I did not recognize it. I just did not know what it was, so I referred him to the dermatologist, who recognized it immediately.’*

### Use of the guideline

The GPs differed in their use of the guideline. On the one hand, it was mentioned that the guideline was difficult to use for deciding whether to order serological testing for LB or not.

‘Very comprehensive. I would like to have a simple flow chart (…) It is about probabilities, that makes it difficult’

On the other hand, the guideline also provided arguments for simply never ordering tests for LB.

‘I read about the test characteristics and then I decided, what I thought before but… then I decided: Okay, I will never test again, serology, I mean. If I consider LB, I just refer.’

### Serological testing in patients with clinical suspicion of LB

The GPs were aware that serological testing is not necessary for patients with a typical EM and that these patients should be treated.

‘When I see an EM, I treat it; then I do not need to perform tests.’

However, serology was sometimes ordered to confirm LB in a patient with EM and treatment was prescribed without waiting for the results of the test.

‘Officially you are not supposed to test for Borrelia, but I do it anyway. Well, yes, you want to be sure.’

The GPs differed in the strategies they used for confirmation of disseminated LB. Some did not order serological tests when they suspected disseminated LB and found that testing in these cases is the responsibility of the medical specialist.

‘I think, if you really think of disseminated LB you should refer. (…) Then I leave it to the medical specialist whether or not he will do serological tests.’

In contrast, some GPs felt confident in diagnosing LB and ordered serological tests themselves.

‘A man with severe pain in his arm. He had a history of a tick bite. In that case, I have ordered serology. (…) I have also treated him myself, by infusion.’ (…) Well, this is a high incidence area, and we get lectures from the internist or microbiologist in the hospital each year. I suppose that makes that we are more alert and that we also treat it ourselves.”

### Serological testing for LB in patients without clinical suspicion of LB

The GPs mentioned different testing routines in patients with non-specific symptoms without a tick bite. One reason for not ordering tests was the perceived high probability of false-positive results.

‘Well I must say, I prefer not to test very often, because, in my view, it is not very helpful with all these very nonspecific complaints, because often you test positive without actually having LB’

Another reason to refrain from testing was referral to of patients with non-specific symptoms who worried about LB to medical specialists:

‘People with complaints, who are worried about LB, I believe that an internist is the most competent doctor to say if it is LB or not. Moreover, my testing does not add sufficiently to that. Because also if it is negative, the uncertainty remains.’

The GPs mentioned several reasons for testing in patients with non-specific symptoms and a low or very low probability of LB. Tests were ordered on patients’ request and to reassure patients who feared to have LB and who were difficult to convince that it is not necessary to test.

‘There was a mother of a fifteen-year-old girl who was tired, exhausted, and with a headache, and she could not concentrate. And yes, the mother thought that she needed to be checked for LB. I tried to explain very kindly that it is not useful. (…). But, oh well, sometimes you do things to maintain the relationship. (…) Of course, it was negative.’

Besides, LB tests were included in routine blood testing in patients with continuing unexplained symptoms, with or without an evident risk of tick bites. The reason behind this was that medical specialists also include LB tests in their analysis of patients with non-specific symptoms.

‘(I request): *leucocytes, CRP, vitamin D, TSH, creatinine, glucose, BSE, haemoglobin, ALAT. And together with that Borrelia serology, celiac disease*.” (…) *Well, yes, I have learnt if you refer them, they do these tests.’*

### Strategies for dealing with the limited accuracy of the serological tests

The GPs in our study were aware of the risks of obtaining false-negative test results in patients with EM and false-positive results in patients with non-specific complaints or without symptoms. All agreed that patients with a typical EM required treatment, irrespective of the test results if serology was ordered.

They applied different strategies to deal with potential false-positive results in case of a (very) low probability.

One strategy included explaining the limitations of the test results on beforehand, ruling out LB if the result is negative and referring patients with a positive result to a medical specialist for further analysis.

‘They may be tested, that is not a problem for me if that is what they want. However, I explain that it does not provide a 100% guarantee whether they do or do not have it. And if the results are positive, they will be referred to an internist for further analysis and if results are negative, we must assume that they don´t have it.’

Another strategy was hoping for negative results, ruling out LB and in case results are positive, explaining that this is likely to be caused by an infection in the past and does not require treatment. Because of difficulties in convincing patients that positive results were not alarming, this strategy could also lead to referral or to prescription of probably unnecessary treatment.

‘Yes, testing makes it difficult. You hope it will be negative, but it is positive. (…) then you try to explain that it is not a recent infection (…) but he remains worried. So, I referred him to the Lyme specialist.’

‘Once I saw a gardener, who had been bitten by ticks many times, and he had some nonspecific complaints, and he said: it must be LB. Then we did serology. (…) It did not seem to be an active infection. Then he wanted to have more analyses, and he went to one of these private laboratories. (…) And the result was that he might have LB. So then, I have treated him, after all. (…) I think I may have treated a resolved LB.’

## Discussion

### Main findings

GPs felt confident in recognising and treating EM, but identifying disseminated LB can be difficult for GPs. However, patients with severe or continuing unexplained symptoms were referred to medical specialist, whether the GP suspected LB or not. The GPs were aware of the guideline for testing, but experienced difficulties applying it. Not in agreement with the guideline, GPs ordered tests in patients with a very low probability of LB. This was done to rule out LB on patients’ request, to reassure worried patients and as part of routine work-up. In these cases, the GPs considered the limited accuracy of the tests. Some GPs applied a clear strategy referring all patients who tested positive; others sometimes struggled explaining possible false positive results.

### Strengths and limitations

This was the first qualitative study about diagnostic practices for LB in general practice in the Netherlands, and as far as we know in Europe. The strength of our study is that with the qualitative design, we have been able to identify reasons behind the results of the quantitative studies about diagnostic practices of LB [3,13]. Using a purposeful sampling strategy, we have been able to find a broad spectrum of diagnostic strategies and explanations for decisions regarding LB. Nevertheless, we may not have identified all strategies used by Dutch general practitioners concerning LB. The GPs in our study, however, were very open describing cases in which they had not followed the guidelines and in which they had not recognised LB. Therefore, it seems unlikely that reporting bias has affected our results.

### Agreement with existing literature

The Dutch guideline is developed for the Dutch health-care system, but internationally the available guidelines do not much differ on the topic of testing. All recommendations for testing are based on prior chances of having LB; minor differences exist in the follow-up of positive and negative test results [15–17]. This qualitative study helps to explain the findings of the two quantitative studies that demonstrated non-adherence to the guidelines for serological testing for LB in the Netherlands [3,13]. The reasons for not following the guidelines that we found, to give in to patients’ requests and to reassure patients, have been reported in a study from the USA [11].

Overutilisation of laboratory tests, testing on patients’ demand and testing to reassure patients have been described in studies concerning unexplained non-specific symptoms [18]. In fact, Dutch GPs consider testing on patients’ request time saving and frequently order blood tests for non-specific symptoms [19,20]. For interpretation of the results of these tests, they generally take into account their pre-test estimation of the probability that the patient suffers from a specific condition [21]. This underscores our finding that the GPs did not routinely explain a positive test result with the diagnosis LB, but considered false-positive results. Fear for LB was mentioned in our study as a reason for patients to request testing. A population-based study in the Netherlands reported that people tend to underestimate their personal risk of getting LB but that they perceive LB as a serious disease [22].

Non-adherence to guidelines because of patients’ preferences is not unique for LB. In a systematic review, patients’ preferences were identified as an essential reason for intentional non-adherence to guidelines [23]. The GPs in our study, who tested on patients’ request to reassure patients often did so considering the limitations of the tests and acted accordingly upon results. Although this testing strategy is not in agreement with the LB guideline, it is recognised as a valid strategy in the Dutch GP guideline of medically unexplained complaints, but in these cases, the GP should explain on beforehand his/her expectation of a negative result and the meaning of a positive result, as is done by some of the respondents [24].

### Implications for clinical practice

The current guideline for testing for LB appears not to align with the questions concerning LB that GPs are confronted with. An algorithm for testing including not only evidence-based indications but also reassurance of patients with a very low probability of LB, together with recommendations for how to act upon results might be helpful. GPs may consider referring patients with non-specific symptoms with positive test results to a medical specialist or a Lyme expertise centre to prevent inappropriate diagnosis and treatment. Because disseminated LB is difficult for GPs to recognise and patients are referred based on alarming or continuing symptoms, rather than on suspicion of LB, medical specialist play a vital role in recognising LB.

## Conclusion

Recognition and diagnosis of disseminated LB can be difficult for GPs, but patients with alarming symptoms related to LB are likely to be referred to medical specialists. GPs do not adhere to the guidelines in ordering serological tests in patients who are unlikely to have LB to reassure patients. In these cases, Interpretation of test results can be difficult.
